# Study protocol: Artificial Intelligence Health Education Accurately Linking System for enhancing self-management in chronic obstructive pulmonary disease patients via WeChat

**DOI:** 10.3389/fmed.2026.1782018

**Published:** 2026-03-24

**Authors:** Ju Wu, Yun-Hua Li, Tingting He, Yan Guo, Jing Zhong, Lifen Wang, Zhen Xiao, Ping Wang, Ping Li, Xia Shu, Qiuhong Guo, Qing Yuan, Lijuan Qiu, Ni Yang, Liping Liao

**Affiliations:** 1 Department of Respiratory and Critical Care Medicine, Zigong First People’s Hospital, Zigong, China; 2 School of Education, Chengdu College of Arts and Sciences, Chengdu, China; 3 School of Nursing, Henan University of Science and Technology, Luoyang, China

**Keywords:** artificial intelligence, chronic disease, chronic obstructive pulmonary disease, large language model, mobile health, RCT

## Abstract

**Background:**

Chronic obstructive pulmonary disease (COPD) is a widespread chronic illness that affects millions of people around the world. Its management not only necessitates medical intervention but also long-term patient self-management. Most traditional self-management education fails to address individualized needs and ongoing support. With improvements in information technology, especially in the application of artificial intelligence (AI) for health management, new avenues have emerged that can improve COPD patients’ self-management capacity, ultimately leading to better disease control and improved quality of life. Thus, we have developed an AI Health Education Accurately Linking System (AI-HEALS) to examine if AI-HEALS-based intervention is effective in enhancing COPD patients’ ability for self-management, ultimately leading to better disease management and improved quality of life.

**Methods and analysis:**

This randomized controlled trial (RCT) aims to evaluate the efficacy of AI-HEALS intervention implemented through WeChat platform in improving self-management ability of COPD patients. Eligible COPD patients meeting the inclusion criteria will be randomly assigned to either an intervention group or a control group. The control group will receive standard treatment, while the intervention group will receive the AI-HEALS program in addition to standard treatment. The AI-HEALS system is an AI driven question and answer platform with integrated voice interaction capabilities, designed to facilitate easier interaction between patients and the system. It provides real-time physiological monitoring, medication and behavior reminders, and personalized health education through the “COPD Health Management Expert” WeChat platform. The system is supported by a self-developed knowledge base based on established clinical guidelines and evidence for COPD, ensuring the accuracy and reliability of the information provided. Outcome measurements will be conducted at multiple time points, including baseline (upon enrollment), hospital discharge, the end of the intervention, and at 3, 6, and 9 months after the completion of the intervention.

**Ethics and dissemination:**

Ethical approval for the study was obtained from the Medical Ethics Committee of Zigong First People’s Hospital [Ethics No. Ethics (Research) 2024, Issue 152, 31/10/2024]. The clinical trial is registered under ChiCTR2400092829, with registration completed on 25/11/2024.

**Study Protocol Registration:**

[https://www.chictr.org.cn/bin/home], identifier [ChiCTR2400092829].

## 1 Introduction

Chronic obstructive pulmonary disease (COPD) is one of the most common respiratory disorders, primarily characterized by progressive airflow limitation, which may be accompanied by inflammatory lung diseases (1, 2). COPD is highly prevalent globally, with a global prevalence of 10.3% (95% CI 8.2–12.8) among people aged 30–79 years in 2019, based on GOLD case definitions, according to the results of a systematic review (3). The major risk factors for COPD include long-term smoking, air pollution, occupational exposures, and genetic predisposition (4). COPD not only has a profound impact on individual health, leading to persistent dyspnea, fatigue, and frequent respiratory infections, but it also imposes a substantial burden on families and society (5). For example, disease exacerbations often result in a decline in the patient’s ability to work, increasing the caregiving burden on family members (5). Additionally, frequent hospital visits and long-term medical expenses place considerable strain on healthcare systems (6).

Currently, the treatment of COPD primarily relies on pharmacological therapy, rehabilitation training, and patient education (1, 2). Pharmacological treatment mainly includes long-acting β2-agonists, long-acting muscarinic antagonists, and corticosteroids (7). These medications help alleviate symptoms, improve lung function, and reduce the frequency of acute exacerbations (7). Additionally, pulmonary rehabilitation, which includes aerobic exercise, strength training, and breathing techniques, has been shown to enhance patients’ exercise tolerance and quality of life (8). Patient education, on the other hand, aims to increase patients’ understanding of the disease and improve their self-management abilities, thereby enhancing symptom control.

Although these traditional interventions have been somewhat effective, they exhibit several notable limitations. First, their reliance on face-to-face interactions and in-person treatment settings has resulted in unequal distribution of healthcare resources and limited access to medical care, particularly in low-resource and remote areas (9). Moreover, medication adherence and long-term maintenance pose additional challenges. Many patients may interrupt their treatment due to side effects, cost concerns, or misunderstandings about the effectiveness of the therapy. Furthermore, low participation rates in pulmonary rehabilitation are a common issue, with many patients lacking motivation to engage in rehabilitation programs, or facing barriers such as limited facilities or trained personnel (10). Lastly, existing patient education often overlooks the integration of mental health support and behavioral health, which are crucial for promoting health behavior change and managing COPD in the long term (11).

In recent years, mobile health interventions have also demonstrated significant potential in the management of COPD. A systematic review by Zangger et al., which included 130 randomized controlled trials, demonstrated that digital health interventions significantly improve objective measures of physical activity and function in COPD patients compared to traditional care (12). These interventions also resulted in improvements in depression, anxiety, and health-related quality of life (12). Additionally, a study by Zanaboni et al., involving the random allocation of 120 participants, found that remote rehabilitation and unsupervised training effectively reduced the incidence of hospitalizations and emergency visits, while significantly improving health status and exercise capacity over the course of 1 year (13). Further confirming these findings, Wang et al. showed that interventions delivered via mobile health applications not only significantly improved scores on COPD Assessment Tests and self-management scales over a 12-months period, but also promoted exercise and smoking cessation behaviors in patients (14). However, despite these encouraging findings, there are still certain limitations to current mHealth solutions for COPD that need to be addressed. Currently, mHealth solutions for COPD are largely based on standardized and rule-based designs, which are not very adaptable to individual variations and needs. Indeed, current mHealth solutions for COPD are largely unidimensional, focusing only on specific aspects such as exercise encouragement or monitoring of symptoms, and do not provide comprehensive mHealth solutions for long-term self-management of these patients. Additionally, since current mHealth solutions do not make use of intelligent data analysis and active decision-making, they are not very effective at making timely and personalized interventions to these patients.

In today’s social arena, application of the Artificial Intelligence Health Education Accurately Linking System (AI-HEALS), an artificial intelligence-based health education system on WeChat, the largest social media in China, is especially significant. AI-HEALS uses economical and ubiquitous mobile communication technology, which enables patients to easily obtain ongoing disease care and management from home. Interventions in AI-HEALS are based on the integrative Health Behavior Promotion Model (HAPA-MTM), which not only supports physical symptoms management in patients but also focuses on mental health and behavior change, ultimately providing an all-encompassing and long-term management approach (15, 16). Its main features involve AI-based Q&A based on an in-house knowledge database, physiological indicator tracking and behavior monitoring, various reminder services, and customized information delivery. This new intervention model is more adaptable to cater to health management requirements of contemporary society, with an effective support and management solution for COPD patient population, and demonstrating the potential of emerging technology in health management.

In summary, this study proposes to assess the efficacy of AI-HEALS intervention in managing one’s own health in COPD patients by conducting a randomized controlled trial (RCT), with special emphasis on the following:

- Evaluate the efficacy of a 3-months intervention program based on the AI-HEALS in enhancing physiological markers in COPD patients;
- Assessing the efficacy of a 3-months multidisciplinary intervention program based on AI-HEALS to improve psychological markers among COPD patients;
- Assess the efficacy of a 3-months multifaceted intervention program based on the AI-HEALS to enhance self-management behaviors among COPD patients.

## 2 Materials and methods

### 2.1 Design

**FIGURE 1:**
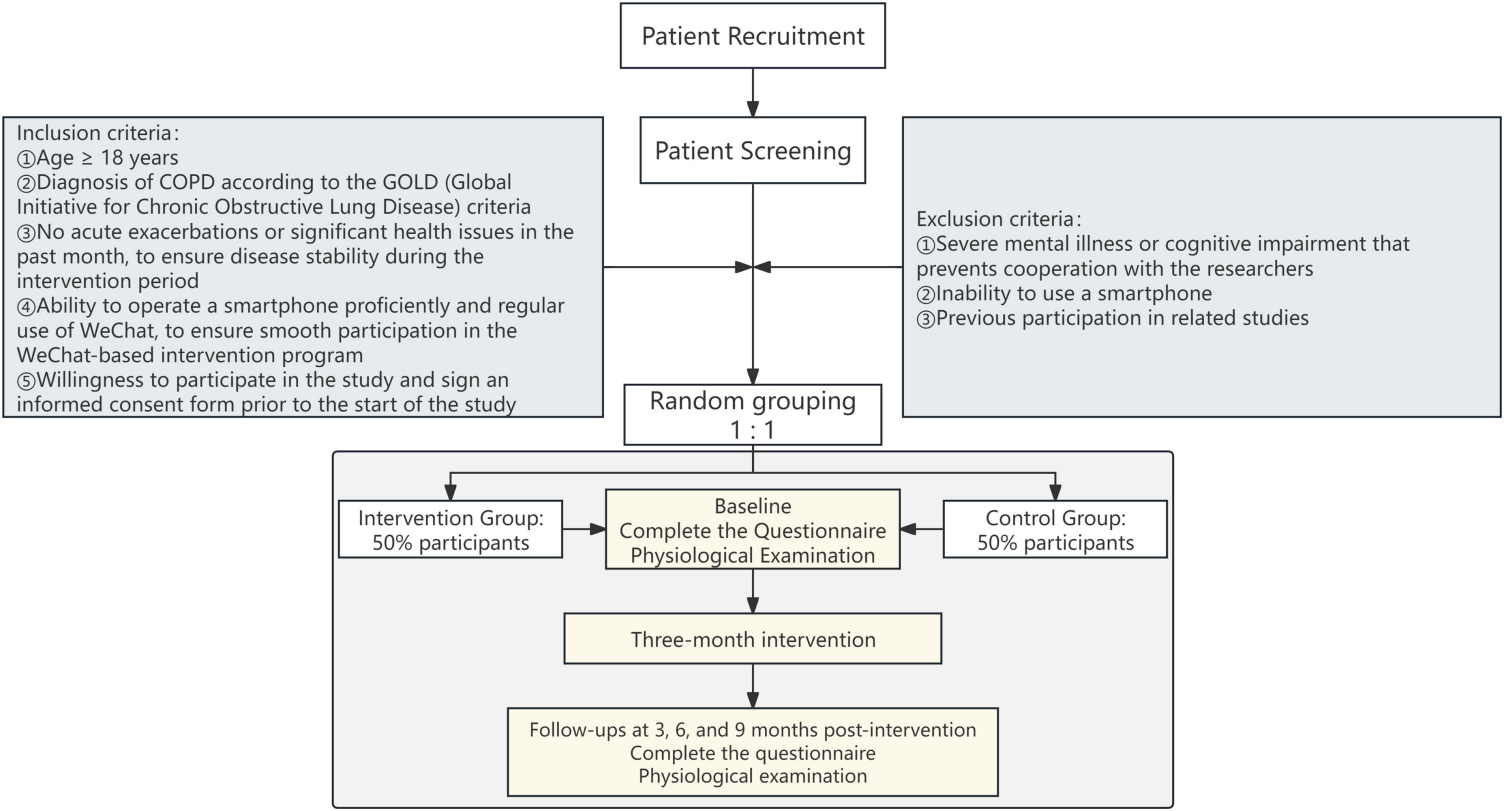
Flow chart of patient recruitment and study implementation. Eligible patients with chronic obstructive pulmonary disease are screened and randomized in a 1:1 ratio to the intervention or control group. Baseline assessments are conducted prior to the intervention. The intervention group receives a 3-months AI-HEALS–based program in addition to standard treatment, while the control group receives standard treatment alone.

It is a single-blind, parallel-controlled, randomized study in which patients diagnosed with COPD from a general public hospital in Zigong, China, participated. This is a single-center study. The study design and grouping are presented in Figure 1.

### 2.2 Randomization and blinding

In this research, a computer-generated random number sequence will be applied in randomization. Patients will be assigned to intervention and standard care groups in a 1:1 ratio. Randomization will be carried out prior to their hospital admission. Through this process, every patient will be assigned to their corresponding study group based on a predetermined sequence to ensure randomness and equity of allocation. Upon entering their ward, each patient will receive a unique code that is equivalent to the random number sequence for facilitation of smooth and uninterrupted recruitment. Once randomization is completed, patients will be transferred to their respective ward depending on their assigned groups. Specifically, intervention patients will be admitted to Ward A, with patients in the control arm going to Ward B to avoid possible cross-contamination between different treatments.

In order to maintain confidentiality of sequence allocation, an independent team member not involved in participant registration or in intervening in any way in the allocation process will perform randomization. This step aims to avoid revealing the randomization sequence during the study. The allocation outcome will then be communicated by the independent team member to the intervention team so that neither the participant registration team nor intervention team knows beforehand against which group a participant is registered.

Finally, due to the novelty of this AI-HEALS intervention therapy in this trial, full participant and therapist blinding is not possible. This limitation might lead to some type of biases, such as performance bias due to participants’ knowledge of their treatment allocation, or detection bias due to knowledge by therapists of implemented treatments. To mitigate these biases, we will take a series of steps to mitigate their effects on the study. Firstly, participant names of all intervention and control groups will be kept anonymous until study completion. Secondly, an independent study staff member who does not take part in participant registration or intervention delivery will be responsible for conducting the random allocation. This individual will forward the allocation results to the intervention team directly to prevent both the enrollment team and intervention team from knowing group assignments beforehand. Thirdly, intervention group patients will be admitted to Ward A, while patients in the control group will stay in Ward B, to avoid any cross-contamination between intervention and control groups. Lastly, to further decrease bias, study data will be collected by research assistants without knowledge of their group allocations. Further, during the analysis process, no knowledge of group allocations will exist for data analysts. During conducting the study, data will be coded and kept confidential to preserve the integrity of blinding.

### 2.3 Study sample

All patients diagnosed with COPD are eligible to participate in the study. Additionally, participants must meet the following criteria:

#### 2.3.1 Inclusion criteria

(1) Age ≥ 18 years; (2) Diagnosis of COPD according to the GOLD (Global Initiative for Chronic Obstructive Lung Disease) criteria; (3) No acute disease exacerbations or any severe health conditions over the past month, ensuring stability of disease status over the intervention duration; (4) Proficiency in operating a smartphone and use of WeChat on a regular basis, to facilitate easy participation in WeChat-based intervention program; (5) Being willing to take part in the study and sign an informed consent agreement before starting the study.

#### 2.3.2 Exclusion criteria

(1) Severe mental illness or cognitive impairment that hinders collaboration with the researchers; (2) Inability to operate a smartphone; (3) Prior involvement in similar studies.

#### 2.3.3 Withdrawal criteria

(1) Individuals withdraw from participation in the study due to personal reasons such as scheduling conflicts or change of interest; (2) Development of severe adverse effects in relation to study participation, including acute event (for example, myocardial infarction, severe infection) or severe illness that necessitates suspension of intervention; (3) Alteration in participant status or medical advice that requires adjustment of the initial treatment regimen, which would be in conflict with study intervention.

### 2.4 Sample size

This RCT will be conducted in two sequential phases. The first phase serves as a pilot study aiming to obtain preliminary estimates of the intervention’s effect size, variability, and feasibility parameters. These pilot data will provide empirical inputs for calculating the appropriate sample size required for the subsequent full-scale RCT.

In this pilot phase, the primary objective is not hypothesis testing but to estimate outcome variance and effect size with sufficient precision to inform future sample size determination. Following commonly recommended practices for pilot RCTs (17), approximately 25 participants per group (about 50 in total) will be recruited. Considering an anticipated 10%–20% attrition rate, the total recruitment target is set at 60 participants (30 per group).

The effect size derived from this pilot phase will then be used to conduct a formal power analysis to determine the optimal sample size for the main RCT, ensuring adequate statistical power to test the study hypotheses.

### 2.5 Recruitment

This study aims at enrolling participants who qualify for inclusion and exclusion in the target hospital. Recruitment will be performed by the medical staff of the hospital, which will screen eligible patients upon their discharge or admitting them to extend their invites. Next, an initial personal contact with patients will be made by the research team, whose study’s purpose, procedures involved, probable gains, and possible risks will be explained in details. Eligible patients will be invited to their respective hospitals for a comprehensive health evaluation, which includes an in-depth review of the history of COPD, and symptoms. Upon ensuring that patients understand the study completely by answering their questions, participants will be asked to sign an approved consent document. Participants that have signed an approved consent document will be officially enrolled in a study. Apart from this, we will use a consistent measurement proxy to make sure that measurements by self-report are reliable.

### 2.6 Informed consent

The process of informed consent will be done openly and transparently, adhering strictly to ethics. Participants meeting inclusion and exclusion criteria will be given a detailed written document describing the aims of the study, procedures, possible hazards and gains, and measures for protecting their information. It also includes explicit details of their information being collected, stored, and secured. We will keep all data securely encrypted on secured servers, accessible only by authorized members of our research team during the study, and properly disposed of after study completion. Participants will have sufficient time to read carefully through the consent document, request any questions, and provide consent voluntarily. Participants will be clearly informed that they can withdraw from the study at any point without any adverse consequences. We will offer continuous points of contact for consultations so that participants can seek additional information or clarifications if required. During the study, we will make all new information available to participants that could change their decision to remain in the study, ensuring continuous informed consent.

In addition, individuals who withdraw from the intervention group will not be penalized. Where intervention is underway, patients who withdraw cannot be reassigned to the control arm and will be counted as a natural attrition case. Semi-structured interviews with patients who withdraw, where possible, will be conducted to learn about their reasons for withdrawal. Participants will be made explicitly informed of all possible options upon withdrawal at the point of informed consent, so that they are aware of their rights and choices at all times.

### 2.7 Intervention

#### 2.7.1 Control group: standard treatment group

Participants assigned to the control group will receive routine medical care provided by a multidisciplinary medical team, which follows established clinical guidelines for the management of COPD. Standard nursing includes: (1) regular outpatient visits, treatment plans formulated by physicians, and clinical evaluations; (2) Follow up at predetermined intervals to monitor the condition; (3) Conduct routine diagnostic evaluation based on clinical indications (such as lung function testing and measurement of basic physiological indicators); (4) Provide traditional health education during clinical contact, with a focus on disease awareness, medication adherence, and lifestyle recommendations; (5) If necessary, refer to relevant specialist physicians.

The control group participants will not be able to use the AI-HEALS system, nor will they be able to access AI based digital health education, personalized reminders, or interactive self-management support provided through the WeChat platform. Except for the lack of AI-HEALS intervention measures, the two groups remained consistent in other aspects of routine clinical care.

#### 2.7.2 Intervention group: standard treatment + AI-HEALS-based COPD online health education program

**FIGURE 2:**
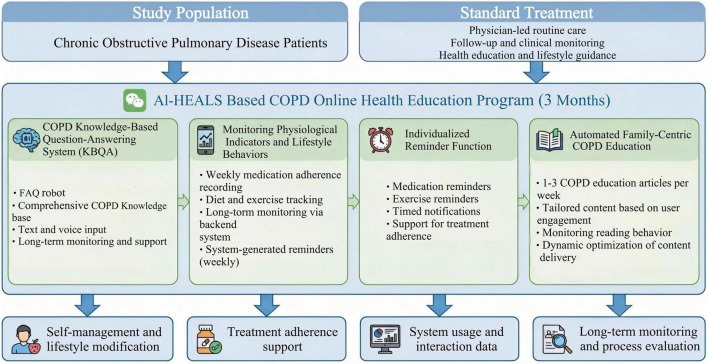
Study design and technical pathway of the AI-HEALS. All participants receive standard physician-led routine care. Participants in the intervention group additionally receive a 3-months AI-HEALS–based online health education program delivered via WeChat, which includes a COPD knowledge-based question-answering system, monitoring of physiological and lifestyle indicators, individualized reminders, and automated COPD education to support self-management and treatment adherence.

**FIGURE 3:**
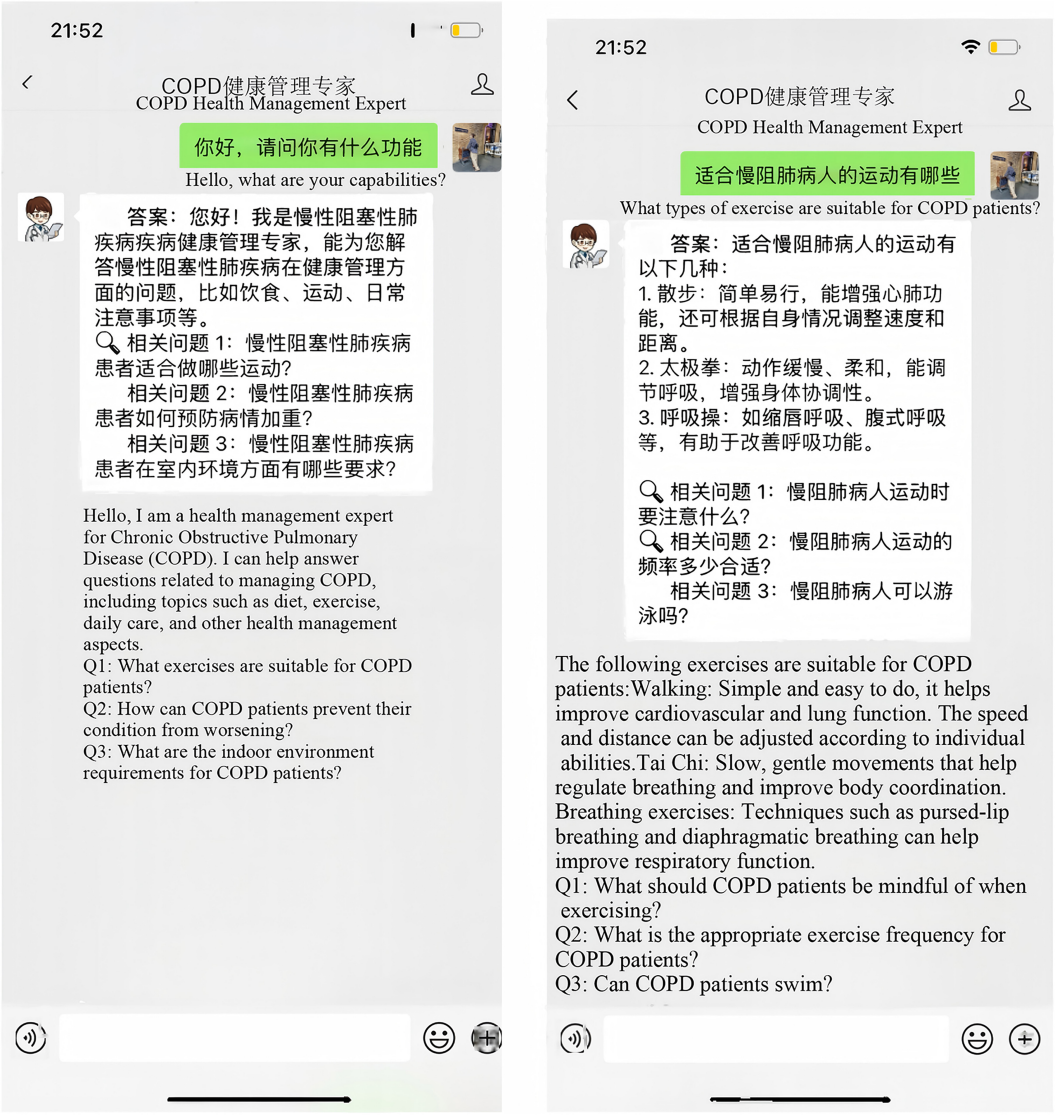
Artificial Intelligence Health Education Precision Linkage System responses to chronic obstructive pulmonary disease knowledge. Representative screenshots show text-based interactions between patients and the AI-HEALS system via the WeChat interface, illustrating guideline-based responses to common COPD self-management questions and the provision of context-relevant follow-up prompts.

In accordance with settings for earlier RCTs in COPD, this trial used a 3-months intervention. Throughout these 3 months, AI-HEALS will administer the intervention to the intervention arm via the “COPD Management Expert” WeChat application. Public health professionals, clinicians, clinical nurses, psychologists, and statistician analysts make up the intervention team with their individual sets of expertise contributing to intervention development. Their intervention program is based on four main components which are as follows (Figure 2):

Chronic obstructive pulmonary disease Knowledge-based Question Answering System (KBQA): the COPD KBQA is designed to support patient health education by providing accurate and guideline-concordant information related to COPD. The system is implemented as a frequently asked questions (FAQ) robot embedded within the WeChat platform (Figure 3), enabling patients to conveniently access COPD-related educational content through text or voice interaction. A structured and self-developed COPD knowledge base underpins the KBQA system. The knowledge base was constructed based on established clinical guidelines and evidence-based literature and covers key domains including disease concepts, etiology, symptoms, pharmacological and non-pharmacological treatments, medication use, daily self-management strategies, dietary recommendations, and appropriate physical activity. To ensure clinical accuracy and safety, the content of the knowledge base is reviewed and periodically updated by clinical experts with experience in COPD management. The KBQA system integrates this curated knowledge base with Doubao, a ByteDance-developed large language model (LLM), which serves as a natural language interface to facilitate patient–system interaction. The LLM was not fine-tuned with COPD-specific data. Instead, it is configured to generate responses grounded in the predefined COPD knowledge base, thereby constraining outputs to validated educational content rather than unrestricted free-text generation. This design aims to enhance the reliability and safety of the information provided while maintaining conversational usability. To promote sustained engagement, the system automatically recommends three context-relevant follow-up questions after each user query. User interaction data, including question content, access frequency, and usage time, are securely logged at the backend for system optimization purposes. These data are analyzed to identify common informational needs and to guide iterative refinement of the knowledge base under expert supervision. The KBQA system is intended solely to support health education and self-management and does not provide diagnostic or treatment decision-making advice.

Recording and Tracking Physiological Signs and Lifestyle Modifications: AI-HEALS will be utilized in this trial to encourage COPD patients to track regularly lifestyle components, such as their compliance with medications each week, their diets, and exercise. Participants will be able to enter information directly into the system, with researchers gaining instant access to recorded material via a backend interface, allowing long-term tracking and monitoring of self-management. In order to establish relevance and accuracy of data, the research team will work with families to create SMART (Specific, Measurable, Achievable, Relevant, Time-bound) plans through initial participation. Data collection will then be framed against these plans with system-generated reminders every Saturday to facilitate uploading of information. This will enable overall evaluation of intervention effects on patients’ everyday life as well as ensuring that data is aligned with individual-family-related targets.

**FIGURE 4:**
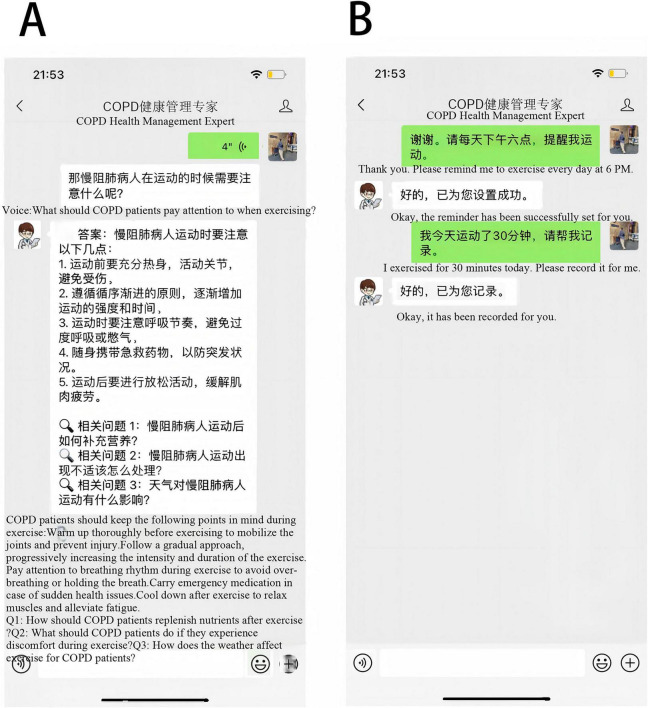
Artificial Intelligence Health Education Precision Linkage System. **(A)** Voice interaction; **(B)** Reminder and record features. Panel **(A)** demonstrates voice-based interaction for accessing COPD health education content. Panel **(B)** illustrates automated reminders and behavior-recording functions, such as exercise reminders and confirmation of completed activities, designed to support long-term self-management.

Individualized Reminder Function: to facilitate treatment compliance, the AI-HEALS system will provide an individualized reminder service (Figure 4). Participants can conveniently set reminders on specific tasks, e.g., taking medicine and exercise. Once reminders are programmed, notifications will be sent by the system at designated times. Through this reminder function, patients are expected to comply strictly with medical treatment advice, ensure long-term continuation of healthful behaviors, and ultimately help them better manage COPD.

Automated Family-Centric COPD Education Articles: in addition, there will also be an automated system for dispensing education articles. COPD patients will receive one to three COPD management-related articles every week that cover different topics. Examples of topics include nutrition tips on which foods are good for COPD patients to eat and which foods to avoid, exercise tips that provide appropriate levels of physical activity, and precise drug information such as optimal administration procedures and which expected side effects to watch for. Educational content will be distributed in a tailored way to address each participant’s individual requirements based on their engagement with the system, such as the types of questions submitted, frequency of visits, and areas of increased interest. To ensure optimum use of information dissemination, background information, such as number of documents read by participants, their preference of type of documents, and length of time spent on each document, will be continuously monitored. Frequency of distribution and content will be optimized based on these findings.

Lastly, in the context of this research, maintaining participants’ privacy and guaranteeing security of their information in their engagements with the AI-HEALS system is imperative. Patient information that is collected using the AI-HEALS system will be handled under maximum confidentiality and will only be utilized for research. Each of our members in the research team is under binding privacy and confidentiality agreements, which means that they are bound by law and ethics to protect sensitive information from participants. In addition, study participants are entitled to withdraw from participation unconditionally at any point without any explanation. This right ensures that participants’ autonomy is respected and that no participant is made to proceed with their participation. Finally, all intervention-related adverse events will be carefully documented and reported in strict compliance with local law and in accordance with standard protocols.

#### 2.7.3 Strategies to improve adherence to interventions

This study will ensure participant compliance with the following three strategies. (1) While recruiting participants and seeking their signed consent forms, researchers will clarify in great detail the participant requirements for the duration of observation and establish a good relationship with them to gain their trust. (2) Throughout the trial, participants will be educated in a thorough way about disease management and preventive practices. This involves educating them on the nature of their illness, why it’s important to stick with treatment, and steps to avoid any potential complications. By educating participants about their own care, we hope to engage better with medical advice and actively participate. (3) The intervention group’s interaction with the system on the portal will be monitored by the study team weekly to review their engagement.

### 2.8 Outcomes

#### 2.8.1 Primary outcomes

The primary outcome is the change in CAT scores (COPD Assessment Test), measured at baseline, discharge, and post-intervention.

#### 2.8.2 Secondary outcomes

Changes in self-management behaviors: including diet, physical activity, medication adherence, smoking management, alcohol management, sleep management, quality of life, and activities of daily living.

Social Cognition and Psychological Factors: COPD knowledge literacy, self-efficacy, depression, anxiety, stress, and social support.

Control of physiological indicators: cough, breathlessness, blood pressure, height, and weight.

30-days readmission rate: additionally, recruitment rates, dropout rates, and reasons for withdrawal will be collected. To assess the homogeneity of the data, demographic characteristics and outcomes of those who dropped out, as well as those with resistance to the trial, will be compared during the data analysis process.

### 2.9 Variables measurement

Sociodemographic variables will be obtained at baseline. Physical examinations and questionnaires will be completed at baseline, discharge, and end of intervention, and at 3, 6, and 9 months post-intervention (Table 1).

**TABLE 1:** The schedule of enrollment, interventions, and assessments.

Study period	Recruitment	Intervention		Follow-up		
Timepoint	0	Discharge	3 months	3 months	6 months	9 months
Eligibility screen	√					
Informed consent	√					
Allocation	√					
Sociodemographic variables	√					
CAT	√	√	√	√	√	√
mMRC	√	√	√	√	√	√
CET	√	√	√	√	√	√
PHQ4	√	√	√	√	√	√
PSSS-SF	√	√	√	√	√	√
NGSES-SF	√	√	√	√	√	√
EQ-5D-5L	√		√	√	√	√
Smoking	√		√	√	√	√
Alcohol consumption	√		√	√	√	√
B-PSQI	√		√	√	√	√
FCS-SF	√					
eHEALS	√					

B-PSQI, The new Brief Version of the Pittsburgh Sleep Quality Index-Short Form; CAT, COPD Assessment Test; CET, Cough Evaluation Test; eHEALS, The eHealth Literacy Scale; EQ-5D-5L, EuroQol-5 Dimensions 5 Levels; FCS-SF, The Family Communication Scale-Short Form; IPAQ-SF, The International Physical Activity Questionnaire Short Form; mMRC, Modified Medical Research Council; PHQ-4, the abbreviated Patient Health Questionnaire-4; PSSS-SF, The Perceived Social Support Scale; NGSES-SF, The New General Self-Efficacy Scale-Short Form; SREBQ, The Self-Regulation of Eating Behavior Questionnaire.

After discharge, we will maintain contact with participants via WeChat, telephone, or text messages to ensure they stay well-informed and remain actively engaged. During these interactions, participants will receive timely updates on study progress, solutions to any emerging issues, and timely reminders for upcoming follow-up assessments.

Standardized data entry for this study will be conducted using an electronic data capture (EDC) system. First, double data entry will be performed by two independent operators to ensure accuracy, followed by logical validation checks on all raw data. Sensitive information will be encrypted using the Unicode encoding scheme. Data will be stored on password-protected servers with role-based access control (where only authorized researchers can access de-identified data), and a real-time backup system will be deployed to off-site cloud storage to ensure data integrity and disaster recovery capabilities.

Once we have obtained relevant information from participants, we will take instant steps to remove all identifiable personal details in an effort to maintain confidentiality of data. Throughout this process, identifiable personal details will be coded with a unique code. This key connecting such codes to real identity details will be accessible by only a few authorized members of our research team and will be kept in a distinctly separate location. We will never provide any personal details to third parties until such time that we receive explicit, informed consent from the participant. All members of the research team will be required to enter into confidentiality agreements that will help guarantee that every team member maintains the highest standards of privacy. Throughout the study from beginning to end, and after it is finished, all personally identifiable information will be stored securely on specified servers that only officially designated researchers can access.

Socio-demographic variables: gender, age, marital status, location, average household size, educational level, occupation, as well as clinical information, including the degree of lung function and detailed medical history.

Anthropometric variables: height, weight, waist circumference, and blood pressure will be measured twice for each participant using certified instruments. Height and weight will be measured using fully calibrated, certified electronic devices. The body mass index (BMI) will be calculated by dividing weight (in kilograms) by height (in meters squared). Waist circumference will be measured at the level of the umbilicus using a flexible tape measure. Blood pressure will be measured using a validated automatic blood pressure monitor to assess both systolic and diastolic pressures.

Respiratory function: it will be assessed using a series of pulmonary function tests (PFTs) that provide insights into lung mechanics and gas exchange, including the percentage of forceful expiratory volume predicted in 1 s (FEV1) and FEV1/forceful lung volume (FVC) (18). FEV1 indicates the volume of air exhaled in the first second after administration of bronchodilators, whereas the FEV1/FVC ratio indicates the degree of airway obstruction.

### 2.10 Questionnaires

CAT: the COPD Assessment Test is used to assess the impact of COPD on patients’ health and daily life (19), covering eight aspects: cough, sputum production, chest tightness, activity level, ability to perform daily activities, social activities, sleep, and energy levels. Each question is scored on a 6-point scale (0–5), with the total score ranging from 0 to 40. Based on the total score, COPD severity is categorized as follows: 0–10 (mild impact), 11–20 (moderate impact), 21–30 (severe impact), and 31–40 (very severe impact).

Modified Medical Research Council (mMRC) Dyspnea Scale: the mMRC scale is primarily used to assess the degree of dyspnea in COPD patients (20). It categorizes the severity of shortness of breath based on the level of activity at which it occurs, with 5 levels (0–4). A score of 0–1 indicates minimal symptoms, scores of 2 and above indicate more frequent symptoms, and a score of 4 represents the most severe dyspnea, where the patient experiences difficulty breathing even with minimal exertion.

Cough Evaluation Test (CET): the Cough Evaluation Test consists of five items (21), including “daytime cough severity,” “impact of cough on sleep,” “severity of coughing episodes,” “impact of cough on daily activities,” and “anxiety caused by coughing.” The CET uses a Likert scale for scoring, with each item rated from 1 to 5, yielding a total score range of 5–25. A higher score indicates more severe cough symptoms or greater impact.

The assessment of depression and anxiety will be conducted using the Patient Health Questionnaire-4 (PHQ-4). This is a brief self-report tool designed to evaluate an individual’s depression and anxiety symptoms over the past 2 weeks (22). The PHQ-4 consists of four items, which assess two dimensions: the first two items focus on depressive symptoms, while the latter two address anxiety symptoms. Each item is rated on a four-point scale: “Not at all” (0 points), “Several days” (1 point), “More than half of the days” (2 points), and “Nearly every day” (3 points), with a total score range of 0–12. The PHQ-4 demonstrates high validity and reliability, with a Cronbach’s alpha coefficient of 0.833 in research studies conducted in China.

The Perceived Social Support Simplified Scale (PSSS-SF) is designed to assess an individual’s perception of social support (23). The scale consists of three items, each corresponding to a different dimension of support: family support (Item 1), friend support (Item 2), and other forms of support (Item 3). Each item is rated on a 7-point Likert scale, ranging from 1 (strongly disagree) to 7 (strongly agree), with a total score ranging from 3 to 21. A higher score indicates a greater perception of social support, reflecting the effectiveness of the individual’s social network in providing emotional and practical assistance.

The New General Self-Efficacy Scale (NGSES-SF) is used to assess an individual’s confidence in their ability to successfully complete specific tasks or overcome challenges (24). This scale also consists of three items, which measure three dimensions of self-efficacy: the level or degree of capability (Item 1), the strength of confidence (Item 2), and the generalizability of this confidence (Item 3). Responses are rated on a 5-point Likert scale, with scores ranging from 1 (strongly disagree) to 5 (strongly agree), and the total score ranges from 3 to 15. Higher scores directly reflect a greater level of self-efficacy, indicating stronger confidence and coping ability.

The EQ-5D-5L scale (EuroQol-5 Dimensions 5 Levels) will be used to assess quality of life (25). This scale specifically measures five key health dimensions: mobility, self-care, usual activities, pain/discomfort, and anxiety/depression. Each dimension is rated on five levels, ranging from “no problems” to “extreme problems,” allowing respondents to select the level that best represents their condition. The responses provide a comprehensive five-dimensional health state profile. In addition to these five dimensions, the EQ-5D-5L includes a visual analogue scale (VAS), which allows respondents to rate their current health status on a scale from 0 to 100 based on their subjective perception of their health.

Smoking behavior will be assessed using a self-designed questionnaire consisting of four multiple-choice questions, aimed at categorizing respondents based on their smoking habits (26). These questions are intended to determine whether the individual currently smokes, how many years they have been smoke-free, their daily smoking consumption, and their age when they started smoking. The first question asks: “Do you currently have a smoking habit?” The response options are: (1) Yes, I smoke regular cigarettes; (2) Yes, I smoke e-cigarettes; (3) I smoke both; (4) I used to smoke (currently quit); (5) I have never smoked. Based on the responses, participants will be classified into two groups: smokers (current smokers) and non-smokers (never smoked or quit).

Drinking behavior will also be evaluated using a self-designed questionnaire, which includes seven multiple-choice questions designed to gain detailed insights into the respondents’ drinking habits. These questions cover aspects such as whether the respondent drinks alcohol, their age at first use, age at cessation, types of alcohol consumed, daily alcohol intake, their average daily consumption before quitting, and any anxiety experienced during the quitting process. The respondents will be asked: “Do you currently drink alcohol, or have you drunk in the past?” The response options are: (1) Never drank; (2) Always drink; (3) Drank in the past but do not drink now; (4) Did not drink before, but now drink.

The Pittsburgh Sleep Quality Index Short Form (PSQI-SF) is a condensed version of the Pittsburgh Sleep Quality Index, specifically designed to assess an individual’s sleep quality (27). This scale consists of six items covering five key dimensions: sleep efficiency (items 1 and 2), sleep onset latency (item 3), sleep duration (item 4), sleep disturbances (item 5), and overall sleep quality (item 6). The sleep onset and wake-up time data are used to calculate sleep efficiency. The total score ranges from 0 to 15, with higher scores indicating poorer sleep quality, reflecting greater sleep difficulties.

The Family Communication Scale Short Form (FCS-SF) aims to assess the effectiveness of communication within the family (28). The scale consists of four items, measuring a single dimension, and uses a 5-point Likert scale ranging from 1 (strongly disagree) to 5 (strongly agree). The total score ranges from 4 to 20, with higher scores indicating better communication ability among family members, reflecting a healthier and more effective communication pattern.

The Chinese eHealth Literacy Scale (eHEALS) is a comprehensive measure to evaluate patients’ integrated knowledge, functional convenience, and perceived ability in searching, assessing, and utilizing eHealth information to solve everyday health-related issues (29). It has 5 items, 1 dimension, with a 5-point Likert scoring system from 1 (strongly disagree) to 5 (strongly agree). The range of total scores is from 5 to 25, with high scores reflecting a better individual eHealth literacy.

### 2.11 Statistical analysis

We will collect quantitative data using a structured questionnaire and then input it into Microsoft Excel for analysis. All major analyses will follow intention to treat (ITT) and will undergo protocol compliant (PP) analysis for sensitivity testing. Descriptive statistics will be used to summarize feasibility variables such as starting information, number of participants, and system usage. For continuous data, the mean and standard deviation of normally distributed data, as well as the median and 25th to 75th percentile of non-parametric data, will be provided. To compare the untreated group and the group measured once, independent sample *t*-tests and their non-parametric analogues will be used. The categorical data will be expressed in frequency and percentage, and analyzed using chi square test. The normality of the data will be tested using the Kolmogorov Smirnov test. Under the assumption of random missing data, established multiple imputation methods will be used in appropriate circumstances to handle the missing data. We will examine and compare the missing and wear patterns between each group to identify potential differences in dropout rates, and conduct sensitivity analysis to compare estimated and complete case outcomes. For the main experiments, the focus will be on using a linear mixed effects model to evaluate the main outcome indicators. For secondary outcomes such as self-management and psychosocial/cognitive outcomes, analysis will use similar mixed effects models or simpler comparison methods, depending on the nature and timing of data collection. In addition, exploratory age stratification analysis can be conducted to examine the potential heterogeneity of intervention effects across different age groups in a descriptive manner. Given the pilot nature of the study, the results should be interpreted with caution. The measurement criteria used by the system, such as the number of questions, will be summarized in a descriptive manner and can be included as covariates in exploratory analysis to better understand compliance patterns; these analyses will be seen as hypothesis generation rather than validation. All statistical tests are two-sided, with a significance level of 0.05. Data analysis will be conducted using IBM SPSS Statistics (version 24.0; SPSS Inc., Chicago, Illinois, USA and Stata (version 14.0); StataCorp, College Station, TX, The United States) conducted.

After the intervention, we will conduct structured interviews with participants to assess the attractiveness, acceptability, usability, and overall satisfaction with the intervention program. These interviews will help explore key factors influencing engagement and behavior change in greater depth. Approximately 30-min telephone or face-to-face in-depth interviews will be scheduled. Demographic variables such as gender, age, location, education level, and severity of COPD will be considered when selecting interview participants. The interview topics will focus on personal experiences, suggestions for improving the AI-HEALS system, motivations for maintaining self-management behaviors, and challenges encountered. To ensure the collection of rich and diverse data, we will continue recruiting participants until data saturation is reached. All interviews will be audio-recorded and transcribed verbatim, ensuring anonymity, and analyzed using a theoretical framework within NVivo 12 (Version 12, QRS International, Doncaster, Australia). Furthermore, qualitative data will be integrated with quantitative data to provide a more comprehensive interpretation of the outcomes at each phase of the trial. This approach will offer a more holistic perspective on the intervention’s effects and its long-term impact on participants.

## 3 Study management

### 3.1 Ethical oversight, data monitoring, and trial governance

A Data Monitoring Committee (DMC) will be established, consisting of at least two members from the Ethics Committee of the First People’s Hospital of Zigong who have no conflict of interest with this study. The committee will conduct routine review and monitoring of research progress and report directly to the ethics committee to ensure its independence from the principal investigator. If any unauthorized deviation from the plan or unauthorized changes are found during the monitoring process, DMC has the right to suspend or terminate the trial.

The hospital ethics committee will conduct a formal review every 6 months to evaluate the progress of the trial and ensure compliance with the approved protocol. All proposed modifications must first be reviewed by the steering committee. After approval, the proposed changes will be submitted to the hospital ethics committee and research department for further review and approval. After approval, a formal document detailing the revised content will be distributed to all relevant parties, and electronic versions will be provided if necessary. All changes to the protocol will be recorded by the trial coordinator and confirmed by the main researcher’s signature. The relevant documents will be archived together with other trial materials. After all approval procedures are completed, the clinical trial registration information will be updated synchronously.

### 3.2 Trial conduct, team communication, and safety monitoring

Prior to trial initiation, the principal investigator (PI) will hold weekly meetings with the trial team to ensure that all study procedures strictly adhere to protocol requirements. During the data collection phase, trial team meetings will be conducted on a monthly basis. In addition, the PI and trial manager will maintain daily communication throughout the study to confirm protocol compliance and address any operational issues promptly.

During the course of the study, if there’s an adverse event (AE) or severe adverse event (SAE), the Ethics Committee must be notified immediately. If the participant suffers from an AE or SAE, with or without relation to the research, the research team must notify the Ethics Committee within 24 h and, if necessary, halt the experiment. In the event of severe errors regarding the AI’s programming and/or severe technological issues regarding the use of the AI, the experiment will be halted instantly. The experiment will only proceed with the redesign and continuation after all the technological issues have been resolved.

### 3.3 Training, protocol adherence, and data quality control

Before the study commences, training workshops and assessments will be conducted by domain experts to ensure that all research team members are fully familiar with study procedures and technical requirements. To minimize loss to follow-up, all participants will be recorded and assigned unique identification codes. To maintain consistency between the intervention and comparison groups, all survey periods will be identical, and the same questionnaires will be administered across all time points.

Data quality will be ensured by carrying out double data entry and verifying the data. The selection of the appropriate analysis procedures would be done after consulting statisticians. If there are any errors discovered in the course of data analysis, the final results would be verified by examining the copies of the questionnaire.

### 3.4 Data management, unblinding, and protocol amendments

Data management will be conducted by a data administrator who will be responsible for administering the recording of the data, cloud storage of the information, accessing the information, as well as solving any problems. The trial administrator will check the information for accuracy.

Unblinding is only permitted under exceptional circumstances, including medical emergencies whereby it is necessary to know to which group the participant belongs. These circumstances include serious adverse events or emergency medical care. The requirement to know group allocations may also be requested following completion of the study for medical reasons. Every request for unblinding will be written by the PI or a member of the clinical review team. A group of professionals will assess the request to determine whether predefined criteria exist for a requested unblinding. Group allocation information will be exchanged as per predefined procedures to maintain participant safety and integrity.

### 3.5 AI system validation, safety controls, and knowledge base updating

Prior to study initiation, the AI-HEALS system will undergo internal validation to ensure the accuracy, consistency, and appropriateness of its educational content. This validation process includes expert review of the COPD knowledge base by clinicians with experience in COPD management, as well as scenario-based testing using common patient questions to verify that generated responses are aligned with established clinical guidelines and predefined educational boundaries.

During the trial, multiple safety control mechanisms will be implemented. The AI-HEALS system is designed to provide health education and self-management support only and does not generate diagnostic or treatment decision-making advice. All responses are constrained to the curated COPD knowledge base to minimize the risk of inaccurate or unsafe outputs. System interaction logs will be monitored regularly by the research team to identify potential anomalies or inappropriate responses, which will be reviewed and addressed in a timely manner.

Procedures for maintaining and updating the knowledge base are established throughout the study period. Updates will be conducted under the supervision of clinical experts and implemented in response to guideline updates, emerging evidence, or identified content gaps. Any modifications to the knowledge base will follow a standardized review process and be documented to ensure transparency and consistency during the trial.

## 4 Dissemination plans

A summary of the RCT findings will be prepared by the research team and emailed to trial participants who indicated interest in receiving the results on their consent forms. The study outcomes will be disseminated through peer-reviewed journal publications and presentations at both national and international conferences. Additionally, findings will be made available upon reasonable request, including to other hospitals for use by medical staff (30, 31).

## 5 Discussion

This study is designed to systematically evaluate the effectiveness of the AI-HEALS intervention, delivered via the WeChat platform, in improving physiological indicators, mental health, and self-management behaviors among patients with COPD through a randomized controlled trial. Although previous interventions have provided some benefits for patients with COPD, long-term self-management remains challenging due to the complexity of the disease, psychological burden, and limited access to continuous, individualized support (9, 10). Existing mobile health interventions have attempted to address these issues. However, their effectiveness has often been constrained by high dropout rates, complex intervention procedures, or a narrow focus on selected behavioral outcomes (12, 13).

Against this backdrop, the present study integrates a 3-months AI-HEALS intervention grounded in the HAPA-MTM framework with a 9-months follow-up period to evaluate both the effectiveness and sustainability of behavior change. The use of artificial intelligence enables the delivery of tailored, timely, and scalable health education, while the integration with WeChat, a widely adopted communication platform, may enhance accessibility and user engagement. By addressing gaps in the literature regarding the long-term sustainability and practicality of digital self-management interventions for COPD, this study aims to contribute empirical evidence supporting the role of AI-enabled tools in chronic disease management.

Several limitations of this study should be acknowledged. First, the single-center design and reliance on the WeChat platform may limit the generalizability of the findings to other healthcare settings, regions, or cultural contexts. Second, due to the nature of the AI-based behavioral intervention, blinding of participants and intervention facilitators is not feasible, which may introduce Hawthorne effects and bias in self-reported outcomes; although standardized procedures and objective indicators are applied where possible, such bias should be considered when interpreting the results. To prevent cross-contamination between groups, participants are allocated to different hospital ward according to their assignments. While this approach helps maintain intervention fidelity, it may introduce potential allocation or environmental bias related to ward-specific contextual factors, despite standardized care protocols across ward. In addition, variations in digital literacy among participants may influence engagement with the intervention, although simplified system features, such as voice input and automated prompts, are incorporated to reduce operational barriers. As digital health literacy is not formally measured, its impact will be interpreted cautiously. Finally, this trial focuses primarily on clinical, psychological, and behavioral outcomes, and does not include formal health economic evaluation. The long-term sustainability of the AI-HEALS system, including cost-effectiveness and knowledge base maintenance, warrants further investigation in future studies.
